# RECIPROCAL RELATIONSHIP BETWEEN FAMILY, FRIENDS, AND COGNITIVE HEALTH AMONG OLDER ADULTS IN CHINA

**DOI:** 10.1093/geroni/igad104.1868

**Published:** 2023-12-21

**Authors:** Xinfang Yu

**Affiliations:** Nanyang Technological University, Singapore, Singapore

## Abstract

Existing research links perceived social support from family and friends to cognitive health in later life. Yet we know less about how cognitive functioning may also shape social support and whether these reciprocal relationships vary between rural and urban areas, especially in China where rural-urban migration is common. This study investigates: (1) whether family support and friendship support help maintaining cognitive functioning, (2) whether cognitive functioning improves perceived family support and friendship support, and (3) whether and how these relationships differ between rural and urban areas. I estimate cross-lagged panel models with a sample including 9,553 adults aged 60 years and above, from two waves of the China Longitudinal Aging Social Survey (CLASS). Results show that cognitive functioning was positively associated with perceived friendship support, regardless of the area of residence. There was a reciprocal relationship between friendship support and cognitive functioning only for rural older adults, with friendship support predicting lower cognitive functioning for urban older adults. Findings suggest that rural and urban context matter – perceived social support, whether from friends or family, may have different implications for the cognitive health of older Chinese adults depending on where they live.

